# Identification of a New Giant Emrbryo Allele, and Integrated Transcriptomics and Metabolomics Analysis of Giant Embryo Development in Rice

**DOI:** 10.3389/fpls.2021.697889

**Published:** 2021-08-09

**Authors:** Zejun Hu, Qiangqiang Xiong, Kai Wang, Lixia Zhang, Ying Yan, Liming Cao, Fuan Niu, Jinyan Zhu, Jinlong Hu, Shujun Wu

**Affiliations:** ^1^Rice Research Center, Crop Breeding and Cultivation Research Institute, Shanghai Academy of Agricultural Sciences, Shanghai, China; ^2^Shanghai Agricultural Products Preservation Processing Engineering Technology Research Center, Shanghai, China; ^3^Innovation Center of Rice Cultivation Technology in Yangtze Valley, Ministry of Agriculture, Jiangsu Key Laboratory of Crop Genetics and Physiology, Co-Innovation Center for Modern Production Technology of Grain Crops, Yangzhou University, Yangzhou, China

**Keywords:** giant embryo, map-based cloning, metabolomics, transcriptomics, metabolic pathway

## Abstract

Rice embryos are rich in high-quality protein, lipid, vitamins and minerals, representing the most important nutritional part of brown rice. However, the molecular mechanism of rice embryo development is poorly understood. In this study, two rice cultivars with contrasting embryo size (the giant embryo cultivar Dapeimi and the normal embryo cultivar 187R) were used to explore excellent genes controlling embryo size, and the developed near-isogenic lines (NILs) (NIL-D, which has the giant embryo phenotype, and its matching line, NIL-X) were used to explore transcript and metabolic properties in the earlier maturation stage of giant embryo development under natural conditions. The map-based cloning results demonstrated that Dapeimi is a novel allelic mutant of the rice *GIANT EMBRYO* (*GE*) gene, and the functional mutation site is a single cytosine deletion in the exon1. A total of 285 differentially accumulated metabolites (DAMs) and 677 differentially expressed genes (DEGs) were identified between NIL-D and NIL-X. The analysis of DAMs indicated that plants lacking *GE* mainly promoted energy metabolism, amino acid metabolism, and lipid metabolism pathways in the rice embryo. Pearson correlation coefficient showed that 300 pairs of gene-metabolites were highly correlated. Among them, *OsZS_02G0528500* and *OsZS_12G0013700* were considered to be key genes regulating L-Aspartic acid and L-Tryptophan content during rice giant embryo development, which are promising to be good candidate genes to improve rice nutrition. By analyzing rice embryo development through a combination of strategies, this research contributes to a greater understanding of the molecular mechanism of rice embryo development, and provides a theoretical foundation for breeding high-nutrition varieties.

## Introduction

Rice is one of the most important food crops in the world, with more than half the global population subsisting predominantly on rice grain (Seck et al., 2012). Rice grain contains abundant starch and cellulose, as well as a wealth of vitamins, trace elements, and bioactive substances. It not only provides the calories required for human productive labor, but also the nutrients necessary for human growth (Babu et al., 2009). With recent improvements in living standards, the demand for increased nutritional quality in rice grains has increased; thus, analyzing the breeding and molecular mechanisms of rice nutritional quality has become an important research area (Fontanelli et al., 2021). Rice grain consists chiefly of the endosperm, embryo, and seed coat, among which 70–80% of nutrients are enriched in the seed coat and particularly the embryo. The endosperm occupies approximately 90% of the rice grain volume, but is enriched with large amounts of starch (Zhou et al., 2002). Consequently, the composition, structure, and properties of the embryo directly determine the nutrition quality of rice grains.

Giant embryo rice, which refers to rice cultivars with larger embryos, whose volume is typically more than twice that of conventional rice, is an ideal material for studying rice embryo development. Moreover, compared with conventional rice, giant embryo rice exhibits greatly increased contents of amino acids, vitamin E, gamma-oryzanol, phenols, and minerals, especially gamma amino-butyric acid (GABA) (Zhang L.L. et al., 2005; Park et al., 2009; Seo et al., 2011; Jeng et al., 2012; Wang et al., 2013; Chen et al., 2020). GABA is a free amino acid that functions as an inhibitory neurotransmitter and typically accumulates in rice embryos. It has multiple physiological functions, such as normalizing blood pressure and aiding recovery from disorders of the autonomic nervous system (DeFeudis, 1983). Using different F2 populations, previous researchers mapped the giant embryo gene on chromosome 7 to a region within 19.2 cM (centiMorgan) and 5.8 cM, respectively (Koh et al., 1996; Qian et al., 1996). Moreover, Dong et al. (2003) suggested that embryo size is a quantitative trait, and genes controlling embryo length and embryo width are located on different chromosomes. Zhang G. H. et al. (2005) performed quantitative trait locus (QTL) analysis on multiple traits of a double haploid population and its parents, and showed that the size of the rice embryo was controlled by genes on chromosomes 3, 4, and 9. At present, *GE* is the first identified giant embryo gene, which encodes the cytochrome P450 designated as CYP78A13/CYP78B5 (Cahoon et al., 2003; Park et al., 2009; Nagasawa et al., 2013; Yang et al., 2013; Chen et al., 2015). CYP78B5 has three conserved domains: an N-terminal leucine-rich region, which is related to membrane localization; a P450 superfamily domain, which contains an oxygen binding site to perform monooxygenation function; and a heme-binding region, which combines with Fe ion to perform an enzyme catalytic function. Chen et al. (2015) showed that CYP78B5 functions in endoplasmic reticulum and peroxisome; loss of its function will cause a sharp decline in the auxin level, affect cell division and expansion, and eventually lead to giant embryo development. The *le* mutant showed mild enlargement in embryo size, which resulted from an increase in the size of scutellar parenchyma cell, and the *LE* gene encodes a C3H4-type RING finger protein (Lee et al., 2019).

Most previous studies on rice giant embryo mutants have focused on genetic analysis and functional research of single gene. Thus, the causal relationship between rice embryo metabolites and related genes has been rarely reported, and few studies have analyzed the relationship between the development regulation mechanism and biomolecular function of rice embryos. In recent years, with the continuous development and improvement of high-throughput sequencing technologies, the integration of multiple omics data has emerged and become a new direction for molecular mechanism research (Moreno-Risueno et al., 2010; Ma et al., 2016; Moschen et al., 2016; Shen et al., 2016; Rai et al., 2017; McLoughlin et al., 2018). Among these technologies, metabolomics and transcriptomics association analysis can achieve co-expression analysis of DAMs and DEGs. Furthermore, combining such analyses with metabolic pathway enrichment, functional annotation, and other biological function analysis can allow key metabolic pathways, key metabolites, and key genes to be identified (Ma et al., 2016; Moschen et al., 2016). Therefore, metabolomics and transcriptomics association analysis is a powerful tool for studying the molecular mechanism of metabolites.

In this study, we characterize the giant embryo rice cultivar Dapeimi. Map-based cloning is conducted to show that Dapeimi is a novel allelic mutant of *GE.* Its exon1 has a single cytosine deletion, which results in a truncated protein of CYP78B5. Furthermore, metabolomics and transcriptomics association analysis reveals that amino acid metabolism, energy metabolism, and lipid metabolism pathways are significantly enriched during embryo development in giant embryo rice. Further analysis revealed that 300 pairs of gene-metabolites are highly correlated. These results provide new insights into rice embryo development, which will help accelerate the breeding of high-nutrition rice.

## Materials and Methods

### Plant Materials and Growing Environments

Dapeimi is a local giant embryo indica rice from Jiangxi Province. In the autumn of 2017, the restoring-line 187R, a normal embryo indica rice, was used as the female parent and crossed with Dapeimi. Then, F1 seeds were harvested in the experimental field of the Life Science College of Fudan University, Shanghai (121°E, 31°N, altitude: 4 m, annual average temperature: 15.8°C, average annual sunshine: 1387 h, annual average evaporation: 1346.3 mm, and average annual rainfall: 1078.1 mm), China. In the same year, F1 seeds were planted in a paddy field in Sanya (109°E, 18°N, altitude: 7 m, annual average temperature: 25.4°C, average annual sunshine: 2287.3 h, annual average evaporation: 1950.7 mm, and average annual rainfall: 1826.5 mm), Hainan Province, China, and F2 seeds were collected in the spring of 2018. The obtained F2 segregating population and its family groups were planted in the same locations mentioned above. In the spring of 2020, after six generations of self-pollination, we developed the following NILs: NIL-D, which carries the Dapeimi allele, and NIL-X, which carries the homologous segment from 187R. The two NILs were planted in the experimental field of school in the summer of 2020.

### Field Experiment and Sampling Strategies

The soil type of experimental field in this study was clay, the tillage depth was 20 cm, and the total amount of nitrogen fertilized was 20 kg/667 m^2^. From the critical leaf-age of productive tillers, low level water (3–5 cm) irrigation was kept until 1 week before harvest. The experimental field was subdivided into three plots, and each NIL separately planted three groups (40 plants per group; 5 rows × 8 columns) in one plot. The row spacing was 12 inches, and the plant spacing was 6 inches. The seedlings were transplanted into experimental field on the 19th of June, about 30 days after germination, and the rice plants grew under natural conditions.

The embryo samples were strictly collected in the middle area of each group on approximately the 15th day after flowering, and three copies (three groups) were collected in one plot for each NIL. Therefore, there were nine copies of samples for each NIL. Among these samples, two copies from each plot, six biological replicates totally, were used for metabolite profiling analysis, and the rest samples, three biological replicates totally, were used for transcriptome profiling analysis.

### Morphological and Agronomic Trait Analysis

Quantitative analysis of agronomic traits, including the 100-grain brown rice weight, endosperm weight, and embryo weight for the two parents, Dapeimi and 187R, were performed using 100 grains at maturity. The resulting values are the means ± SE of three biological repeats. Student’s *t*-test was used to compare significant differences between samples. The related phenotype of transgenic lines was also detected according to the above method.

### Map-Based Cloning

Fresh rice leaves of plants at the grain filling stage were obtained from the two parents, the F2 population, and its family groups. Thirty normal embryo plants and 30 giant embryo plants were randomly selected from the F2 population; the same amount of leaves were mixed to form a normal embryo pool and a giant embryo pool. The genomic DNA of each pool was extracted using the Plant Genomic DNA kit (Tiangen, Beijing, China) and detected by single nucleotide polymorphism (SNP) chip array (RICE6K) to initially locate the target gene. HiScan scanner (Illumina Inc., San Diego, CA, United States) was used for chip scanning, and GenomeStudio software was used for raw data analysis. R platform was used for further analysis, such as genotype identification, comparison and map drawing (R Development Core Team, 2011). Simple sequence repeat (SSR) markers were identified from the Gramene database for grasses^1^. Molecular markers containing polymorphism between two parents were screened, and the genotypes of all markers in the target region were determined for recombinant plants in the F2 population and its family groups. Mapmaker/Exp v3.0 was used for linkage analysis and the Kosambi function was used to calculate the genetic distance (Lincoln et al., 1992). The candidate gene (*OsZS_07T0416900*) in the final positioning interval was amplified separately from the two parents, sequenced, and compared using GENtle software (v1.9.4, Magnus Manske, Cologne, NRW). All the primers used in the current study are listed in Supplementary Table 1.

### Rice Transformation

For *OsZS_07T0416900* over-expression, a 1,578 bp CDS fragment of *OsZS_07T0416900* was amplified from the total RNA of 187R and cloned into pMD19-T, and further inserted into the plant binary vector pCAMBIA1304 (GenBank accession number AF234300) to generate the expression vector pCaMV35S:*OsZS_07T0416900*^187*R*^. This construct was then introduced into the Dapeimi background via *Agrobacterium tumefaciens*-mediated transformation using standard protocols. The transgenic rice plants were further confirmed by PCR detection and direct sequencing.

### Metabolite Extraction and UHPLC-ESI-MS/MS Analysis

Prior to analysis, 100 mg of fresh grain embryos were accurately weighed, and the metabolites were extracted using a 400 μL methanol:water (4:1, v/v) solution with 0.02 mg mL^–1^ L-2-chlorophenylalanine as internal standard. The mixture was settled at −10°C and treated by a high-throughput tissue crusher Wonbio-96c (Wanbo Biotechnology, Shanghai, China) at 50 Hz for 6 min, then followed by ultrasound at 40 kHz for 30 min at 5°C. The samples were placed at −20°C for 30 min to precipitate proteins. After centrifugation at 13000 *g* for 15 min at 4°C, the supernatant were carefully transferred to sample vials. The quality control (QC) sample was prepared by mixing equal volumes of all samples to monitor the stability of the analysis (Doppler et al., 2016).

Chromatographic separation of the metabolites was performed on a Thermo UHPLC (Ultra High-performance Liquid Chromatography) system in Majorbio Bio-pharm Technology Co., Ltd. (Shanghai, China). The mobile phases consisted of 0.1% formic acid in water:acetonitrile (95:5, v/v) (solvent A) and 0.1% formic acid in acetonitrile:isopropanol:water (47.5:47.5:5, v/v) (solvent B). The solvent gradient changed according to the following conditions: from 0 to 3.5 min, 0 to 24.5% B (0.4 mL min^–1^); from 3.5 to 5 min, 24.5 to 65% B (0.4 mL min^–1^); from 5 to 5.5 min, 65 to 100% B (0.4 mL min^–1^); from 5.5 to 7.4 min, 100 to 100% B (0.4 to 0.6 mL min^–1^); from 7.4 to 7.6 min, 100 to 51.5% B (0.6 mL min^–1^); from 7.6 to 7.8 min, 51.5 to 0% B (0.6 to 0.5 mL min^–1^); from 7.8 to 9 min, 0 to 0% B (0.5 to 0.4 mL min^–1^); from 9 to 10 min, 0 to 0% B (0.4 mL min^–1^) for equilibrating the systems. The sample injection volume was 2 μL and the flow rate was set to 0.4 mL min^–1^. The column temperature was maintained at 40°C. During the period of analysis, all these samples were stored at 4°C. The mass spectrometric data was collected using an AB Sciex TripleTOF 5600^TM^ mass spectrometer system equipped with an electrospray ionization (ESI) source operating in either positive or negative ion mode with a capillary voltage 1.0 kV, sample cone, 40 V, collision energy 6 eV. The source temperature was set at 120°C, with a desolvation gas flow of 45 L h^–1^. Centroid data was collected from 50 to 1000 m/z with a 30000 resolution.

The acquired MS data from UHPLC-ESI-MS/MS were imported into the Progenesis QI 2.3 (Non-linear Dynamics, Waters, MA, United States) for peak detection and alignment (Nanjegowda et al., 2018). The preprocessing results generated a data matrix that consisted of the retention time (RT), mass-to-charge ratio (m/z) values, and peak intensity. Mass spectra of these metabolic features were identified by using the accurate mass, MS/MS fragments spectra and isotope ratio difference with searching in reliable biochemical databases as Human Metabolome Database (HMDB)^2^ and Metlin database^3^. Concretely, the mass tolerance between the measured m/z values and the exact mass of the components of interest was ± 10 ppm. For metabolites having MS/MS confirmation, only the ones with MS/MS fragments score above 30 were considered as confidently identified. Otherwise, metabolites had only tentative assignments.

### Data Analysis and Validation for Metabolites

A multivariate statistical analysis was performed using R package ropls version 1.6.2^4^. Principle component analysis (PCA) using an unsupervised method was applied to obtain an overview of the metabolic data, general clustering, trends, or outliers were visualized. Orthogonal partial least squares discriminate analysis (OPLS-DA) was used for statistical analysis to determine global metabolic changes between comparable groups. The model validity was evaluated from model parameters R2 and Q2, which provide information for the interpretability and predictability, respectively, of the model and avoid the risk of over-fitting. Variable importance in the projection (VIP) values were calculated in OPLS-DA model. *p*-values were estimated with paired Student’s *t*-test on Single dimensional statistical analysis. Metabolites with VIP ≥ 1.0 and *p*-value ≤ 0.05 were defined as statistically significant. DAMs among two groups were summarized, and mapped into their biochemical pathways through metabolic enrichment and pathway analysis based on database search (KEGG^5^). scipy.stats (Python packages)^6^ was exploited to identify statistically significantly enriched pathway using Fisher’s exact test.

### RNA Isolation and Microarray Analysis

High-quality total RNA was isolated from fresh grain embryos using the PureLink^®^, Plant RNA Reagent kit (Ambion, Austin, United States) according to the manufacturer’s instructions. The RNA concentration was measured using NanoDrop ND-1000 spectrophotometer (NanoDrop Technologies, Wilmington, DE, United States). The purity and integrity of total RNA was determined by 260/280 nm ratio and by NanoBioanalyzer RNA-6000 analysis (Agilent Technologies, Palo Alto, CA, United States). After that, 1μg of total RNA from each sample was used for RNA-Seq library construction following the specifications of the TruSeq^®^, RNA Sample Preparation v2 Guide (Illumina), which was then sequenced using Illumina Hiseq 2500 in Majorbio Bio-pharm Technology Co., Ltd. (Shanghai, China).

The raw paired end reads were trimmed and quality-controlled by SeqPrep^7^ and Sickle^8^ with default parameters. The clean data were assembled using Cufflinks software, then mapped to the reference genome (ZS97RS3, RIGW3.0)^9^ via TopHat with no more than two base mismatches allowed in the alignment (Trapnell et al., 2012). The basic information of the RNA-sequencing data is provided in Supplementary Table 2.

### Data Analysis and Validation for Transcripts

The expression level of each transcript was estimated by the fragments per kilobase of exon per million mapped reads (FPKM) with RSEM (Li and Dewey, 2011). The DEGs were determined using R package edgeR with a false discovery rate (FDR) < 0.05 and a logarithm two-fold change | log2FC| ≥ 1 (Anders and Huber, 2010; Robinson et al., 2010). Gene Ontology (GO) and Kyoto Encyclopedia of Genes and Genomes (KEGG) enrichment analyses were conducted based on the DEGs by Goatools^10^ and KOBAS 2.1.1^11^ (Xie et al., 2011). RNA-Seq data from this article can be found in Gene Expression Omnibus (GEO) under the accession number GSE173301.

### Real-Time Quantitative PCR (qRT-PCR) Based Validation of Genes

Total RNA was isolated as mentioned in the text using the PureLink^®^, Plant RNA Reagent kit (Ambion, Austin, United States) and was reverse transcribed using the M-MLV Reverse Transcriptase kit (Promega, Madison, WI, United States) according to the manufacturer’s instructions. qRT-PCR was performed using the SYBR I Premix ExTaq (Takara Bio, Kusatsu, Shiga, Japan). The gene expression levels in three biological replicates were calculated using the ^Δ^ ^Δ^ Ct (threshold cycle) method (Livak and Schmittgen, 2001). Student’s *t*-test was used to compare significant differences between samples. The primers for selected genes are listed in Supplementary Table 1.

## Results

### Brown Rice Characteristics of Two Rice Cultivars

**FIGURE 1:**
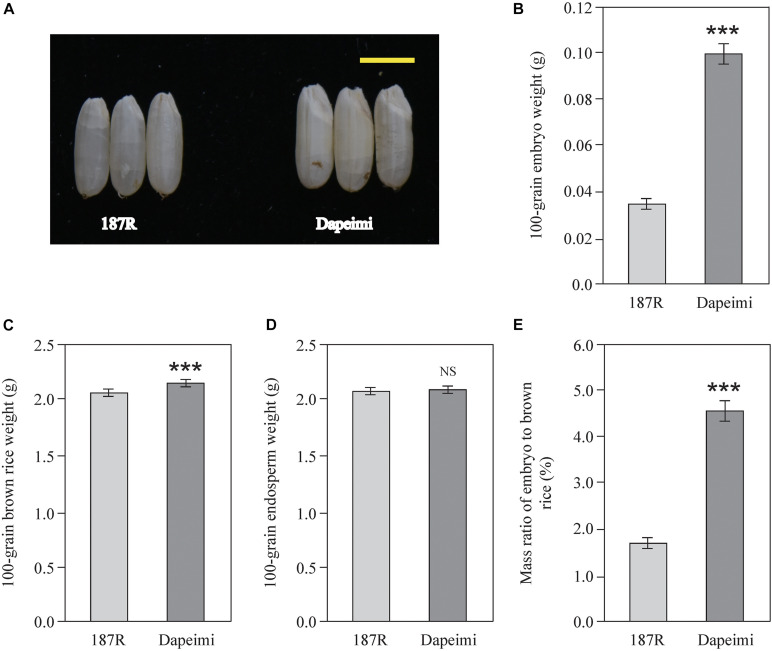
Brown rice phenotypic characteristics of 187R and Dapeimi. **(A)** Brown rice of the parents used for gene mapping analysis. Scale bar, 0.5 cm. **(B–E)** Comparison of embryo weight **(B)**, brown rice weight **(C)**, endosperm weight **(D)**, and mass ratio of embryo to brown rice **(E)** between 187R and Dapeimi cultivars. Data are shown as means ± S.E.M. (*n* = 3). ***P < 0.001; NS, not significant at *P* = 0.05.

Dapeimi is a giant embryo rice cultivar grown in Jiangxi Province (Figure 1A). Its average 100-grain embryo weight was 0.099 g, and the mass ratio of embryo to brown rice was 4.57% (Figures 1B,E). 187R is a derivative line of the India wide-compatibility variety Dular, which has a normal embryo size (Figure 1A). Its average 100-grain embryo weight and mass ratio of embryo to brown rice were 0.036 g and 1.76%, respectively (Figures 1B,E). The two cultivars exhibited significant difference in the 100-grain brown rice weight, but the difference in the 100-grain endosperm weight was not obvious (Figures 1C,D). These data indicate that the size of embryo is the main difference between the two brown rice cultivars.

### Dapeimi Is a Novel Allelic Mutant of GE

**FIGURE 2:**
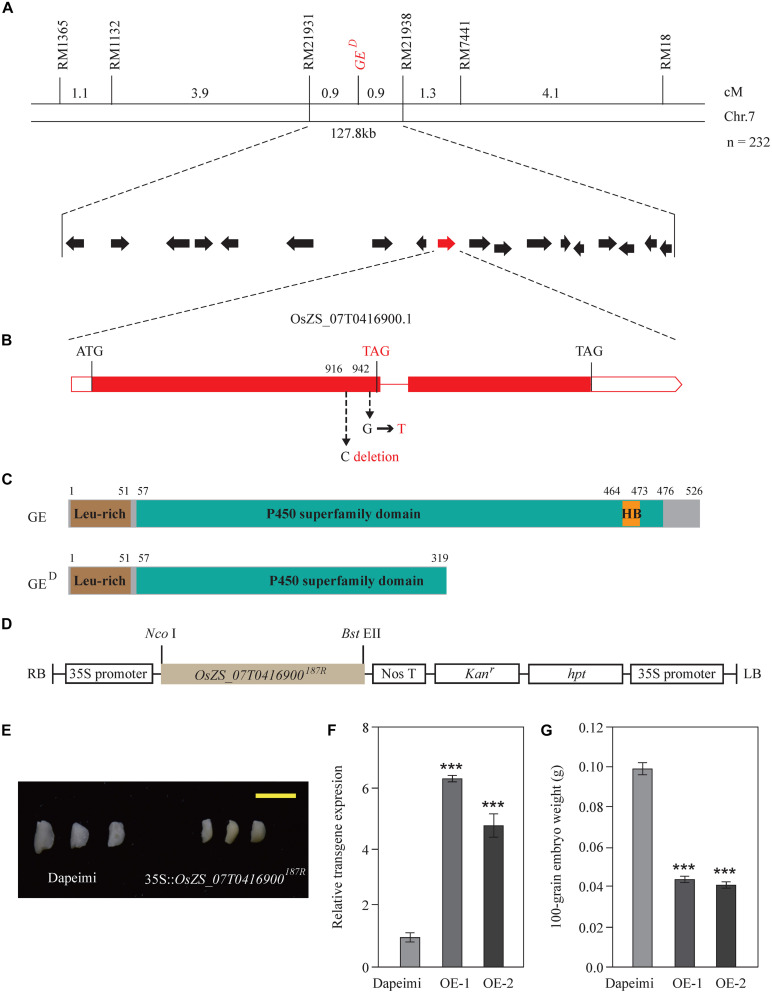
Map-based cloning and confirmation of the *GE*^D^ gene. **(A)** Gene location was performed with 232 giant embryo plants in the F_3_ families. Genetic distance between adjacent markers is indicated above the linkage map. *GE*^D^ locus was narrowed down to a 127.8-kb interval between the SSR marker *RM21931* and *RM21938* on chromosome 7. This region contains 18 predicated genes. **(B)** Gene structure and allelic variations of *OsZS_07T0416900* between 187R and Dapeimi. Filled boxes and red lines represent exons and introns, respectively. Start and stop codons are indicated above the gene, and allelic variations and a premature stop codon are shown in red letters. 187R was used as the reference for allelic comparison. **(C)** Comparison of protein structure between GE and GE^D^. Start and stop positions of each domain are indicated above the protein. **(D)** The basic structure of the expression vector pCaMV35S:*OsZS_07T0416900*^187*R*^. **(E–G)** Phenotypes of embryos **(E)**, transgene expression level of *OsZS_07T0416900* **(F)** and 100-grain embryo weight **(G)** from the control plants (Dapeimi) and the *OsZS_07T0416900* over-expression lines (OE, in Dapeimi background). Scale bar, 0.5 cm. Data are shown as means ± S.E.M. (*n* = 3). ***P < 0.001.

According to the phenotypic investigation, there were 56 giant embryo plants, 60 normal embryo plants, and 92 varied embryo plants in the F2 population derived from Dapeimi × 187R. The segregation ratio agreed with the Mendelian ratio (1:2:1) for single locus segregation (χ^2^ = 2.59 < χ^2^_0__.05_, _2_ = 5.99), which suggests that the embryo phenotypic difference between Dapeimi and 187R was mainly controlled by a single gene. Base on the result of the SNP chip array, the candidate giant embryo gene was roughly mapped on chromosome 7 (Supplementary Figure 1). For fine mapping of this gene, a larger segregating population consisting of 1,000 plants derived from F2 heterozygous plants was constructed. Using 232 giant embryo plants, the locus was eventually narrowed down to a 127.8 kb interval defined by two SSR markers (*RM21931* and *RM21938*). Based on the published sequence annotation for indica rice Zhenshang97 (see text footnote 9), 18 predicted genes were identified in this interval, including the already identified giant embryo gene *GE* (*OsZS_07T0416900*) (Figure 2A). The coding region of *OsZS_07T0416900* was comparatively aligned between Dapeimi and 187R, and a 1 bp base deletion mutation (C deletion) and a non-synonymous mutation (G→T) were detected in the exon1, separately occurring at nucleotide 916 and 942 (Figure 2B). *OsZS_07T0416900* was considered as the favorable candidate gene responsible for the embryo size, because the cytosine deletion mutation in Dapeimi results in a frame shift, leading to premature translation termination of the encoded protein (Figure 2C).

To confirm the mapping results, the expression vector pCaMV35S:*OsZS_07T0416900*^187*R*^ was used to generate *OsZS_07T0416900* over-expression transgenic rice plants (Figure 2D). Ten independent transgenic lines with the Dapeimi background were obtained, and two of them were used for measurements of embryo weight. Most of the 10 independent transgenic lines exhibited decreased embryo size in T1 seeds when compared with wild type (Dapeimi) seeds (Figure 2E), and the decrease in the embryo weight in T1 seeds was up to 60% (Figures 2F,G). These results reveal that disruption of the gene *OsZS_07T0416900* improves embryo size in Dapeimi. Sequence comparison indicated that the mutation type of *OsZS_07T0416900* in Dapeimi does not exist in other giant embryo varieties (Ping et al., 2015). Therefore, Dapeimi is a novel allelic mutant of *GE* with a truncated P450 superfamily domain compared to wild-type rice; we named this gene *GE*^D^.

### Metabolite Profiling of Two NILs

**FIGURE 3:**
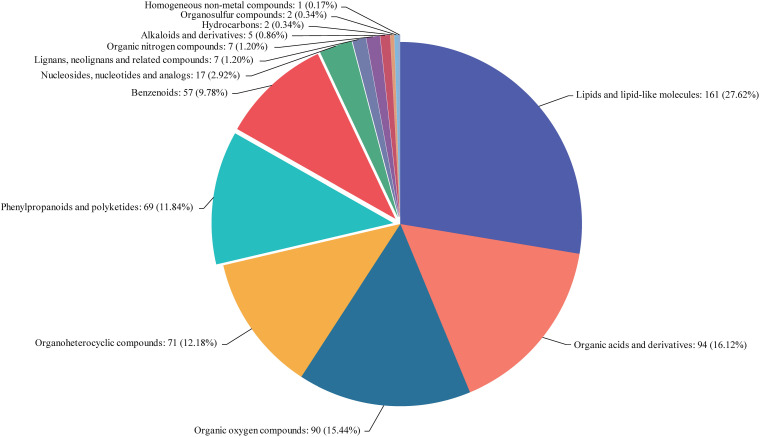
Classification statistics of compounds. Names of the selected HMDB levels (superclass, class, or subclass) and percentage of metabolites are displayed in order from high to low depending on the number of metabolites. Different colors in each pie chart represent different HMDB classifications, whose area represents the relative proportion of metabolites in that classification.

The metabolites in rice embryos from NIL-D (EG) and NIL-X (CG) were investigated using UPLC-ESI-MS/MS. We identified 285 DAMs (148 increased and 137 decreased) from 13 classes. Lipids and lipid-like molecules, organic acids and derivatives, organic oxygen compounds, organoheterocyclic compounds, phenylpropanoids and polyketides, benzenoids, nucleosides, nucleotides and analogs, lignans, neolignans and related compounds, organic nitrogen compounds, alkaloids and derivatives, hydrocarbons, organosulfur compounds, and homogeneous non-metal compounds accounted for 27.62, 16.12, 15.44, 12.18, 11.84, 9.78, 2.92, 1.2, 1.2, 0.86, 0.34, 0.17, and 18.5% of the DAMs, respectively (Figure 3). Detailed DAMs information was shown in Supplementary Table 3. In order to screen metabolites with similar expression patterns, we also conducted the correlation analysis of Top 50 by calculating the correlation and distance between various metabolites and speculating on the function of unknown metabolites (Supplementary Figure 2 and Supplementary Table 4).

**FIGURE 4:**
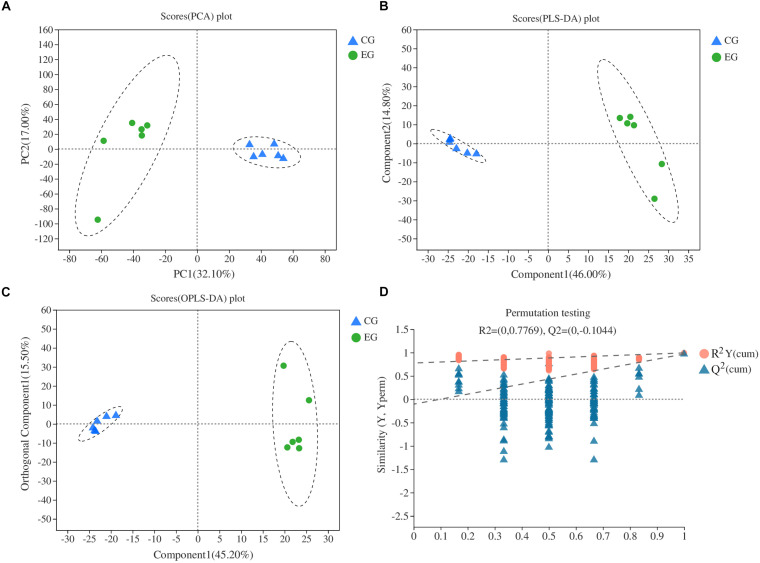
Multivariate statistical score and response-sorting test diagram in two NILs. **(A)** PCA; **(B)** PLS-DA; **(C)** OPLS-DA; **(D)** response-sorting test of the OPLS-DA model. EG, experiment group (NIL-D); CG, control group (NIL-X).

General differences on DAMs between EG and CG samples were revealed using PCA. Two principal components (PC1 and PC2) were extracted and found to represent 32.1 and 17% of the observed variation, respectively. In the PCA score plot, the EG and CG samples were clearly separated, and samples were collected in a compact and repeated way (Figure 4A). PLS-DA demonstrated that the two principal components explained 60.8% of the variation between EG and CG samples (Figure 4B). Moreover, OPLS-DA demonstrated that the two principal components explained 60.7% of the variation between EG and CG samples (Figure 4C). All data points in the figure formed tight clusters between the different comparison groups. To verify the model reproducibility and model fitting, it is imperative to avoid the classification obtained by the supervised learning method. We conducted 200 response-sorting tests of the OPLS-DA model, namely, the fixed X matrix and previously defined Y matrix variable classifications (e.g., 0 or 1) were randomly arranged n times (n = 200). The OPLS-DA model was established to obtain the corresponding stochastic model of R^2^ and Q^2^ values. With the original model of R^2^Y and Q^2^Y linear regression, the regression line and the Y-axis intercept values (R^2^ and Q^2^) were used to measure whether the model was over fitted. The replacement test evaluation standard specifies that the intercept lies between the Q^2^ regression line and *Y*-axis. An intercept of less than 0.05 indicates that the model is robust and reliable, and no overfitting occurs (Figure 4D).

### Transcriptome Profiling of Two NILs

**FIGURE 5:**
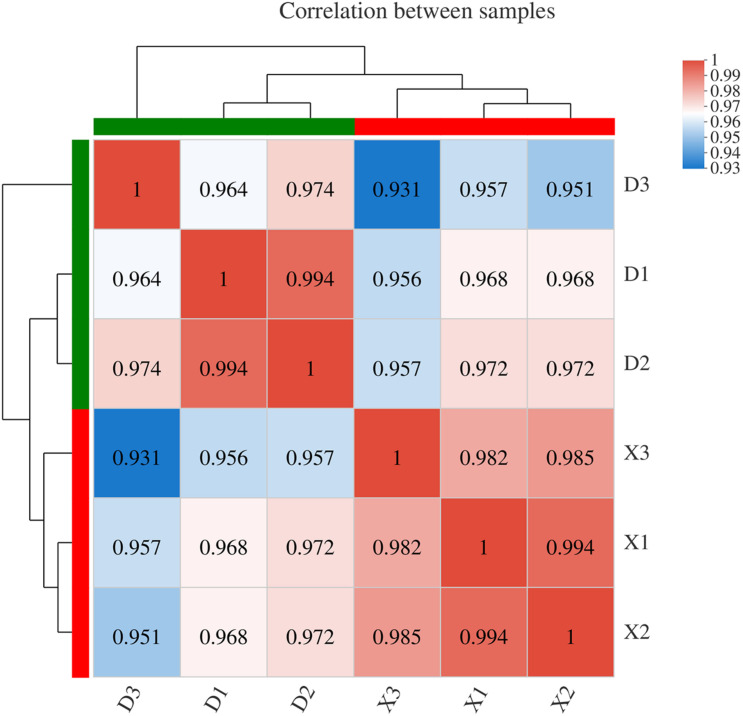
Correlation analysis between different samples. Right and lower sides indicate sample names, X represents three replicates of the CG sample, and D represents three replicates of the EG sample. Left and upper sides are sample clustering, and squares of different colors represent the correlation between the two samples.

To understand the genetic basis for the DAMs observed in EG and CG samples, transcriptome analysis of six samples was conducted in this study. A total of 50.26 Gb of clean data were used, with more than 7.86 Gb of clean data for each sample, and the percentage of Q30 bases was more than 93.03%. A total of 33,861 expressed genes were detected in the analysis, including 32,817 known genes and 1,044 new genes, as well as 64,166 expressed transcripts, including 49,510 known transcripts and 14,656 new transcripts. The genes and transcripts expressed in this study were analyzed by functional database annotation (NR, Swiss-prot, Pfam, COG, GO, and KEGG) (Supplementary Table 2). Three biological replicates were conducted and their repeatability was assayed by calculating the Pearson correlation coefficient (Figure 5). The results were found to be consistent and repeatable. The DEGs were identified between the EG and CG samples using DESeq. Of the EG and CG comparison groups, 677 DEGs were identified, including 313 up-regulated genes and 364 down-regulated genes (Supplementary Table 5). The FPKM value of the validated gene was closely correlated with its relative expression via RT-qPCR (Supplementary Figure 3).

### Metabolic Pathway Analysis of Two NILs

**FIGURE 6:**
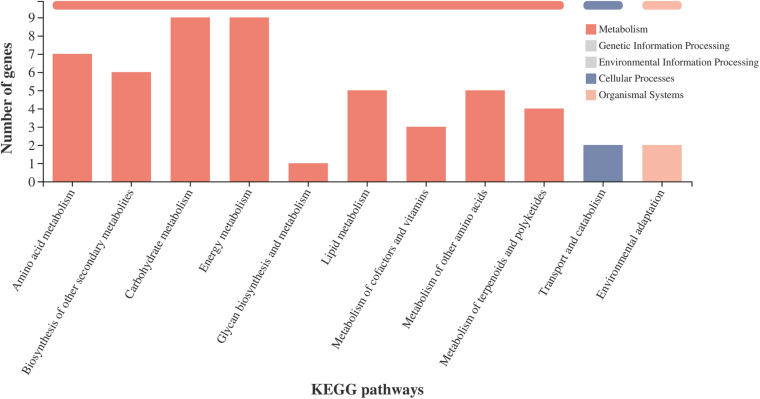
KEGG pathway classification statistics. *X*-axis shows the name of the KEGG metabolic pathway; *Y*-axis shows the number of genes or transcripts annotated to the pathway. There are seven categories of KEGG metabolic pathways: metabolism, genetic information processing, environmental information processing, cellular processes, organismal systems, human diseases, and drug development.

To further investigate the potential functions of the DEGs, we analyzed the metabolic processes. The DEGs in the amino acid metabolism, carbohydrate metabolism, energy metabolism, lipid metabolism, biosynthesis of other secondary metabolites, glycan biosynthesis and metabolism, metabolism of other amino acids, and metabolism of cofactors and vitamins were enriched in the metabolism pathway (Figure 6). Most of the DEGs in the metabolic processes were consistent with the GO enrichment analysis (Supplementary Figure 4). For the second metabolism, the enriched DEGs were correlated with the pathways of lysine degradation, fatty acid degradation, photosynthesis, starch and sucrose metabolism, fatty acid biosynthesis, citrate cycle (TCA cycle), carbon fixation in photosynthetic organisms, flavonoid biosynthesis, amino sugar and nucleotide sugar metabolism, and glycolysis/gluconeogenesis.

### Genes and Metabolites Associated Network Analysis

**FIGURE 7:**
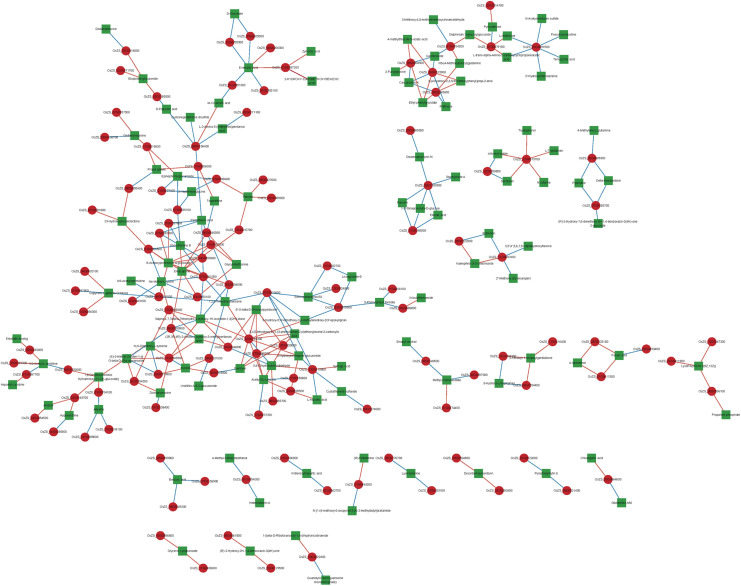
Gene-metabolites associated network analysis. Each node in the figure represents a gene/metabolite, the red square dot represents the gene, the green square dot indicates the metabolites, the size of the node indicates the point connectivity; each connection indicates the interaction between the gene and the metabolites, the red line represents the positive correlation, the blue line indicates the negative correlation, the width of the connection indicates the size of the correlation coefficient, the larger the wider the correlation line.

The various DAMs and genes involved in the significantly enriched amino acid metabolism, energy metabolism, and lipid metabolism pathways of the two NILs are detailed in Table 1 and Supplementary Table 6. To clarify the close correlation of genes and metabolites and screen out the key genes that regulate a certain metabolite, we perform a correlation network analysis of both DEGs and DAMs (Figure 7 and Supplementary Table 7). By calculating the Pearson correlation coefficient of DEG-DAM, it was found that there were 300 pairs of gene-metabolites with the absolute value greater than 0.993, 150 pairs were positive and 150 pairs were negative (Supplementary Table 7). Therefore, these 300 pairs of gene-metabolites were highly correlated.

**TABLE 1:** Candidate DEGs involved in KEGG pathways that are related to amino acid metabolism, energy metabolism, and lipid metabolism pathway.

KEGG pathway	Pathway id	Metabolite	Gene id	FC	Annotation
Cysteine and methionine metabolism	map00270	C00049	*OsZS_09G0262200*	0.049	1-aminocyclopropane-1-carboxylate oxidase 1, putative, expressed
			*OsZS_02G0118500*	103.3	Cysteine synthase, putative, expressed
			*OsZS_01G0494300*	2.312	Serine acetyltransferase protein, putative, expressed
			*OsZS_06G0303600*	2.284	Aminotransferase, classes I and II, domain containing protein, expressed
			*OsZS_09G0196800*	18.51	Expressed protein
Glycine, serine and threonine metabolism	map00260	C00049	*OsZS_09G0304800*	0.263	FAD dependent oxidoreductase domain containing protein, expressed
		C00300			
		C00719			
		C00078	*OsZS_09G0196800*	18.51	Expressed protein
Lysine degradation	map00310	C00449	*OsZS_09G0304800*	0.263	FAD dependent oxidoreductase domain containing protein, expressed
		C00047			
		C04076	*OsZS_02G0528500*	3.186	Aldehyde dehydrogenase, putative, expressed
Lysine biosynthesis	map00300	C00047	*OsZS_09G0196800*	18.51	Expressed protein
		C00049			
		C00449			
		C04076			
		C00666			
Phenylalanine, tyrosine and tryptophan biosynthesis	map00400	C01179	*OsZS_12G0349500*	0.258	Chorismate mutase, chloroplast precursor, putative, expressed
		C00078	*OsZS_06G0303600*	2.284	Aminotransferase, classes I and II, domain containing protein, expressed
Tryptophan metabolism	map00380	C00632	*OsZS_02G0528500*	3.186	Aldehyde dehydrogenase, putative, expressed
		C00955			
		C02693			
		C00643			
		C00780			
		C00078			
		C02470			
beta-Alanine metabolism	map00410	C00049	*OsZS_02G0528500*	3.186	Aldehyde dehydrogenase, putative, expressed
		C00864	*OsZS_04G0535900*	0.402	Amine oxidase, flavin-containing, domain containing protein, expressed
Valine, leucine and isoleucine degradation	map00280	C00407	*OsZS_02G0528500*	3.186	Aldehyde dehydrogenase, putative, expressed
Histidine metabolism	map00340	C00049	*OsZS_02G0528500*	3.186	Aldehyde dehydrogenase, putative, expressed
Phenylalanine metabolism	map00360	C00180	*OsZS_09G0291300*	2.305	caffeoyl-CoA O-methyltransferase, putative, expressed
		C12621	*OsZS_06G0303600*	2.284	Aminotransferase, classes I and II, domain containing protein, expressed
Arginine and proline metabolism	map00330	C00300	*OsZS_04G0007600*	0.255	Pyridoxal-dependent decarboxylase protein, putative, expressed
		C00077	*OsZS_04G0535900*	0.402	Amine oxidase, flavin-containing, domain containing protein, expressed
			*OsZS_03G0445500*	3.445	Expressed protein
			*OsZS_10G0381200*	0.336	Proline oxidase, mitochondrial precursor, putative, expressed
			*OsZS_02G0528500*	3.186	Aldehyde dehydrogenase, putative, expressed
			*OsZS_06G0303600*	2.284	Aminotransferase, classes I and II, domain containing protein, expressed
Pyruvate metabolism	map00620	C03451	*OsZS_12G0142600*	3.893	Expressed protein
			*OsZS_02G0260900*	0.028	Retrotransposon protein, putative, Ty1-copia subclass
			*OsZS_01G0527400*	0.288	Phosphoenolpyruvate carboxylase, putative, expressed
			*OsZS_10G0129500*	4.925	Phosphoenolpyruvate carboxykinase, putative, expressed
			*OsZS_02G0528500*	3.186	Aldehyde dehydrogenase, putative, expressed
			*OsZS_03G0303200*	2.104	Pyruvate, phosphate dikinase, chloroplast precursor, putative, expressed
Arginine biosynthesis	map00220	C00437	*OsZS_04G0419400*	0.483	Glutamate dehydrogenase protein, putative, expressed
		C00049			
		C03406			
		C00624			
		C00077	*OsZS_06G0303600*	2.284	Aminotransferase, classes I and II, domain containing protein, expressed
Alanine, aspartate and glutamate metabolism	map00250	C03406	*OsZS_04G0419400*	0.483	Glutamate dehydrogenase protein, putative, expressed
		C03794			
		C00049	*OsZS_06G0303600*	2.284	Aminotransferase, classes I and II, domain containing protein, expressed
Tyrosine metabolism	map00350	C01179	*OsZS_07G0430000*	0.319	Dehydrogenase, putative, expressed
			*OsZS_06G0303600*	2.284	Aminotransferase, classes I and II, domain containing protein, expressed
			*OsZS_03G0598600*	2.847	Fumarylacetoacetase, putative, expressed
Cyanoamino acid metabolism	map00460	C00407	*OsZS_06G0198100*	0.329	Os6bglu24 – beta-glucosidase homolog, similar to G. max isohydroxyurate hydrolase, expressed
		C00049			
		C15503			
Pentose phosphate pathway	map00030	C00620	*OsZS_02G0412200*	2.129	Glucose-6-phosphate 1-dehydrogenase, cytoplasmic isoform, putative, expressed
Carbon fixation in photosynthetic organisms	map00710	C00049	*OsZS_12G0142600*	3.893	Expressed protein
			*OsZS_03G0303200*	2.104	Pyruvate, phosphate dikinase, chloroplast precursor, putative, expressed
			*OsZS_10G0129500*	4.925	Phosphoenolpyruvate carboxykinase, putative, expressed
			*OsZS_04G0149600*	0.156	Fructose-1,6-bisphosphatase, putative, expressed
			*OsZS_06G0303600*	2.284	Aminotransferase, classes I and II, domain containing protein, expressed
			*OsZS_01G0527400*	0.288	Phosphoenolpyruvate carboxylase, putative, expressed
Galactose metabolism	map00052	C00492	*OsZS_01G0149500*	2.01	UTP–glucose-1-phosphate uridylyltransferase, putative, expressed
			*OsZS_02G0346800*	0.104	Glycosyl hydrolases, putative, expressed
			*OsZS_04G0308800*	0.344	Glycosyl hydrolases, putative, expressed
alpha-Linolenic acid metabolism	map00592	C16343	*OsZS_04G0329700*	0.496	Expressed protein
		C16316			
Linoleic acid metabolism	map00591	C14827	*OsZS_03G0520500*	0.012	Lipoxygenase, putative, expressed
Glycerophospholipid metabolism	map00564	C00670	*OsZS_01G0494000*	3.093	Phosphoethanolamine/phosphocholine phosphatase, putative, expressed
		C04230			
		C04230	*OsZS_05G0437300*	0.146	CPuORF26 – conserved peptide uORF-containing transcript, expressed

## Discussion

### Experimental Design Provides Clues to Reveal the Underlying Molecular Basis of Nutrients Enrichment in Giant Embryo

Giant embryo rice is a special rice characterized by a particularly large embryo. Studies have shown that giant embryo rice is high nutrient functions (Zhao et al., 2019). It is therefore important to studying its molecular basis for breeding high-nutrition rice varieties. However, many previous studies focus on genetic basis of giant embryo rice or nutritional measurements performed on mature rice grains, which lead to little is known about the causal relationship between metabolites and related genes during rice embryo development (Koh et al., 1996; Qian et al., 1996; Dong et al., 2003; Zhang G. H. et al., 2005; Zhang L. L. et al., 2005; Park et al., 2009; Seo et al., 2011; Jeng et al., 2012; Wang et al., 2013; Chen et al., 2020).

Nagasawa et al. (2013) showed that the embryo cell volume in *GE* mutant was more than twice that of the wild-type, but the difference of cell number was not obvious, so the abundance of nutrients in giant embryo rice may be due to the increase of embryo cell volume (the larger the container, the more it could hold). Rice embryo development can be roughly divided into four successive stages: proembryo stage, differentiation stage, maturation stage and quiescence stage. In maturation stage (about 13–25 days), cells basically stop proliferating, embryo still grows slightly at earlier stage, and then is mainly the further accumulation of internal substances (protein, lipids, etc.) (Qin and Tang, 1982). Therefore, we inferred that maturation stage may be the key period for nutrients enrichment in giant embryo rice, and the genes expressed in this period are particularly important. Besides that, the genetic effects of genes on traits in different background could be significantly different, and eventually lead to the deviation of results (Zhao et al., 2017). To correctly evaluate the relationship between genes and metabolites in integrated transcriptomics and metabolomics analysis, the effect of genetic background should be eliminated. For this purpose, NILs are the appropriate materials, which had similar genetic background, but different in a specific trait or genetic basis (Zhang et al., 2009). Here, we characterized genetic basis of giant embryo rice Dapeimi, and analyzed metabolite content and transcript abundance of embryos in the earlier maturation stage of NILs (NIL-D and NIL-X). These offer good opportunities to study the underlying molecular basis of high nutrient functions in giant embryo.

### Energy Metabolism Pathway Involved in Rice Giant Embryo Development

In the process of rice embryo development, maturation stage is an important turning period. Synthesis of starch, as the development signal of this stage, took the lead. Its content increased rapidly from the early differentiation stage, slowed down in maturation stage, and gradually decreased in quiescence stage. The peak value is around the 21st day (Qin and Tang, 1982). Starch plays an important role during rice embryo development, which provides carbon frame and energy for the synthesis of protein, lipid, etc. It is a temporary material for energy storage in rice embryo, that is constantly accumulating and transforming (Qin and Tang, 1982).

**FIGURE 8:**
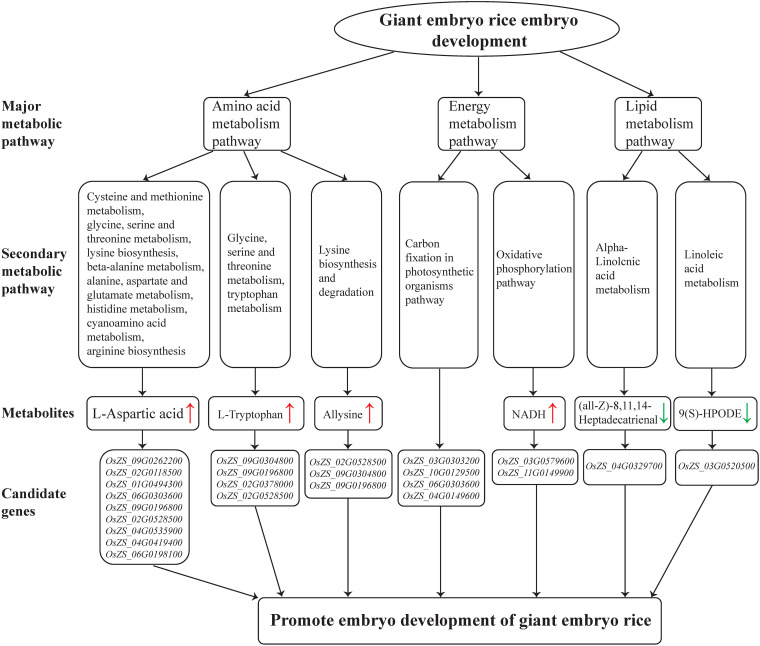
Metabolic pathways involved in rice giant embryo development and candidate genes that regulate metabolites accumulation. Red and green arrows indicate increased metabolites and decreased metabolites, respectively.

According to the metabolomics analysis in this study, energy metabolism, carbohydrate metabolism, and galactose metabolism pathways were significantly enriched in the embryo of NIL-D (Supplementary Table 6). NADH and NAD^+^ are a ubiquitous cellular redox couple, and NAD plays a central role in plant metabolism (Steinbeck et al., 2020). We found that NADH chemicals were significantly increased in the oxidative phosphorylation pathway (Figure 8 and Supplementary Table 6). Besides, carbon fixation in the photosynthetic organism pathway was also significantly enriched (Figure 8 and Supplementary Table 6). Since NADH level is significantly positive correlated with utilization of carbon source (Wan et al., 2018). These imply that giant embryo has a more active anabolism of carbohydrate in earlier maturation stage. The embryo volume of giant embryo rice is 1–2 times greater than that of common rice, and the main components of embryos are protein, lipid, etc. (Azhakanandam et al., 2000; Lee et al., 2019). Therefore, giant embryo rice may need more carbon frame and energy for the synthesis of these substances, and then promoting the accumulation and transforming of energy-storage substances such as starch. Raffinose family of oligosaccharides (RFOs) are the most widespread D-galactose containing oligosaccharides in higher plants (Sengupta et al., 2015). We found that raffinose metabolites decreased significantly in the galactose metabolism pathway (Supplementary Table 6). In orthodox cereal seeds, RFOs are accumulated during latter grain development, and its accumulation is associated with loss of starch in the embryo (Black et al., 1996). It indicates that giant embryo had a lower transforming rate of starch to RFOs in earlier maturation stage. Besides, RFOs accumulation is considered to play an important role for the acquisition of desiccation tolerance during seed development (Sengupta et al., 2015). The ability of seeds to tolerate rapid drying, terminate metabolic activity and survive after rehydration is known as desiccation tolerance, and low desiccation tolerance means low seed vigor (Zhang et al., 2001). Our finding reveals that lower RFOs level maybe an important factor why the germination potential and emergence rate of giant embryo rice are significantly lower than that of corresponding rice.

### Amino Acid Metabolism Pathway Involved in Rice Giant Embryo Development

Relying on the early formation of protein synthesis mechanism and the increasing starch content to provide energy and carbon frame, the content of protein maintains the momentum of growth, even in maturation stage (Zhu et al., 1980). Amino acid is the basic unit of protein, and its metabolism is closely related to energy and carbohydrate metabolism, the carbon-nitrogen budget, and needed for protein synthesis and secondary metabolism (Yang et al., 2020).

According to the metabolomics analysis in this study, amino acid metabolic pathway was significantly enriched in the embryo of NIL-D (Figure 6). In plants, L-Aspartic acid is a precursor for the synthesis of several amino acids, including four essential ones: L-Methionine, L-Threonine, L-Isoleucine and L-Lysine. We found that L-Aspartic acid metabolites were increased significantly in the pathways of Cysteine and methionine metabolism, glycine, serine and threonine metabolism, and lysine biosynthesis etc. (Figure 8 and Supplementary Table 6). Among the candidate genes, *OsZS_02G0528500* was significantly up-regulated (Figure 8 and Supplementary Table 6). Correlation network analysis revealed that *OsZS_02G0528500* was positive correlated with L-Aspartic acid, and the value for Pearson correlation coefficient was 0.996 (Figure 7 and Supplementary Table 7). Thus, it can be inferred that L-Aspartic acid metabolites may be important markers for the giant embryo development, and *OsZS_02G0528500* is a valuable potential candidate gene to regulate L-Aspartic acid content. L-Tryptophan is one of the essential amino acids for human body. It is similar to indole-3-acetic acid (IAA) in structure, and is an important precursor for the biosynthesis of IAA in plants (Zhao, 2018). The main pathway of IAA biosynthesis in higher plants is as follows: L-Tryptophan is firstly oxidized and deaminated to form indole acetone, and then decarboxylated to form indole acetaldehyde, which is finally oxidized to IAA under the catalysis of corresponding enzymes (Zhao, 2018). We found that L-Tryptophan metabolites were increased significantly in the pathways of glycine, serine, and threonine metabolism, and tryptophan metabolism (Figure 8 and Supplementary Table 6). While IAA level was sharply decreased in the seeds of *GE* deficient mutant (Chen et al., 2015). The first step of IAA biosynthesis pathway is reversible. When indole acetone level is too high, the transaminase VAS1 would convert it back to L-Tryptophan (Gao et al., 2016). We speculate that the synthesis of IAA may be inhibited, which leading to the accumulation of upstream metabolites. It may also be due to the different positions and growth periods of materials. Further study is needed to investigate this. Among the candidate genes, *OsZS_12G0013700* was significantly up-regulated (Supplementary Table 5). Correlation network analysis revealed that *OsZS_12G0013700* was positive correlated with L-Tryptophan, and the value for Pearson correlation coefficient was 0.994 (Figure 7 and Supplementary Table 7). Therefore, *OsZS_12G0013700* is a valuable potential candidate gene to regulate L-Tryptophan content. In addition, Allysine metabolites were increased significantly in the lysine biosynthesis and degradation pathway (Figure 8 and Supplementary Table 6). Giant embryo rice is rich in protein and essential amino acids (Zhang L. L. et al., 2005). Our findings of higher L-Aspartic acid, L-Tryptophan, and Allysine level in NIL-D embryos in earlier maturation stage could explain the increased protein and essential amino acids level detected in mature giant embryo rice, providing strong evidence for a causal relationship between amino acid metabolism and protein synthesis.

### Lipid Metabolism Pathway Involved in Rice Giant Embryo Development

Lipid is the third abundant nutrient component of brown rice, mainly distributed in embryo (Wu et al., 2020). Unlike starch, lipid is the ultimate form of energy storage in embryo, and is the main source of energy for rice seed germination. The change of lipid content in embryo is similar to that of starch, but the peak value is around the 15th day (Zhu et al., 1980).

According to the metabolomics analysis in this study, alpha-Linolenic acid metabolism, linoleic acid metabolism, and glycerophospholipid metabolism pathway were significantly enriched in the embryo of NIL-D (Figure 8 and Supplementary Table 6). Specifically (all-Z)-8, 11, 14-Heptadecatrienal, and 13(S)-HOTrE metabolites were down-regulated in the alpha-Linolenic acid metabolism pathway; 9(S)-HPODE metabolite was down-regulated in the linoleic acid metabolism pathway; glycerophosphocholine, LysoPC (15:0), and LPC (16:0) metabolites were down-regulated in the glycerophospholipid metabolism pathway. As mentioned above, giant embryo rice is rich in lipids. Thus, we speculate that these decreased metabolites may be beneficial for the synthesis of lipid in giant embryo. Due to the low lipid content and difficult extraction, the mechanism of lipid regulation in rice embryo is still poorly understood, and more comprehensive and in-depth studies are needed.

## Conclusion

This study revealed that Dapeimi is a novel allelic mutant of *GE*, and the functional mutation site is a single cytosine deletion in the exon1. DAMs analysis of NILs indicated that a lack of *GE* promotes the energy metabolism, amino acid metabolism, and lipid metabolism pathways of rice embryos. In addition, the contents of NADH, L-Aspartic acid, and L-Tryptophan metabolites involved in the key metabolic pathways increased, whereas the raffinose content decreased. Further correlation network analysis of DAMs and DEGs screened out 300 pairs of gene-metabolites that highly correlated in the earlier maturation stage of rice embryo development. According to their functional annotations, we preliminary determined two potential candidate genes, *OsZS_12G0013700* and *OsZS_02G0528500*, for the content regulation of L-Aspartic acid and L-Tryptophan respectively. These findings can be used to provide genetic resources for the future cultivation of giant embryo rice with high nutritional value, and is significant to improve the nutritional value of endosperm and develop new rice varieties through synthetic biological methods. The functional identification of genes in metabolic pathways related to rice embryo development is in progress. Finally, we plan to construct some relevant vectors and express these genes in the rice endosperm in order to improve its nutritional value.

## Data Availability Statement

The datasets presented in this study can be found in online repositories. The names of the repository/repositories and accession number(s) can be found below: NCBI GEO, accession no. GSE173301.

## Author Contributions

ZH and QX performed most of the experiments. KW, LZ, and YY developed the rice mapping populations and NILs. LC and FN helped to map the gene. JZ and JH contributed to analysis of the data. ZH, QX, and SW designed the experiments, analyzed the data, and wrote the manuscript. All authors have discussed the results and contributed to the drafting of the manuscript.

## Conflict of Interest

The authors declare that the research was conducted in the absence of any commercial or financial relationships that could be construed as a potential conflict of interest.

## Publisher’s Note

All claims expressed in this article are solely those of the authors and do not necessarily represent those of their affiliated organizations, or those of the publisher, the editors and the reviewers. Any product that may be evaluated in this article, or claim that may be made by its manufacturer, is not guaranteed or endorsed by the publisher.

## Footnotes

1
http://www.gramene.org/

2
http://www.hmdb.ca/

3
https://metlin.scripps.edu/

4
http://bioconductor.org/packages/release/bioc/html/ropls.html

5
http://www.genome.jp/kegg/

6
https://docs.scipy.org/doc

7
https://github.com/jstjohn/SeqPrep

8
https://github.com/najoshi/sickle

9
http://rice.hzau.edu.cn/rice_rs3/

10
https://github.com/tanghaibao/Goatools

11
http://kobas.cbi.pku.edu.cn/download.php

## Abbreviations

NIL: near-isogenic line
GE: GIANT EMBRYO
DAM: differentially accumulated metabolite
DEG: differentially expressed gene
cM: centiMorgan
SNP: single nucleotide polymorphism
SSR: simple sequence repeat
QC: quality control
UHPLC: ultra high-performance liquid chromatography
RT: retention time
HMDB: Human Metabolome Database
m/z: mass-to-charge ratio
PCA: principal components analysis
OPLS-DA: orthogonal partial least-squares-discriminant analysis
VIP: variable importance in the projection
FPKM: fragments per kilobase of exon per million mapped reads
FDR: false discovery rate
GO: gene ontology
KEGG: Kyoto Encyclopedia of Genes and Genomes
GEO: Gene Expression Omnibus
qRT-PCR: real-time quantitative PCR
^Δ Δ^ Ct: threshold cycle
EG: experiment group
CG: control group
RFO: raffinose family of oligosaccharide.
