# Supplementary material for: Identification of a New Giant Emrbryo Allele, and Integrated Transcriptomics and Metabolomics Analysis of Giant Embryo Development in Rice
Source: Front Plant Sci. 2021 Aug 9;12:697889. doi: 10.3389/fpls.2021.697889 (PMC8381154; doi:10.3389/fpls.2021.697889)
Supplement: Supplementary file 1 [file Data_Sheet_1.docx]

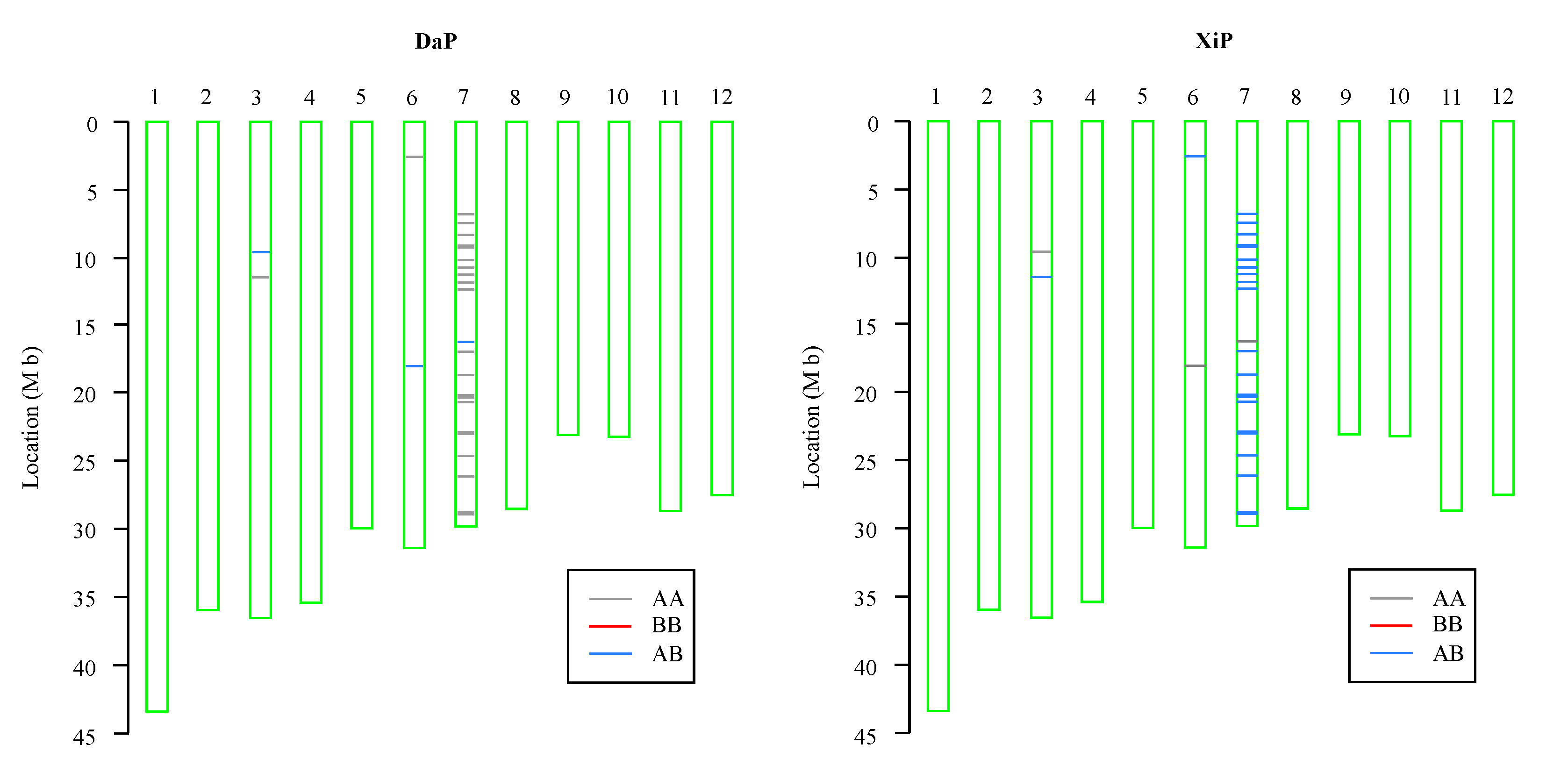


**Figure S1.** SNP chip localization using BSA mixed pool analysis. The giant embryo and normal embryo genotype are indicated by letters AA and BB separately, AB represents heterozygous genotype.
