# Supplementary material for: Identification of a New Giant Emrbryo Allele, and Integrated Transcriptomics and Metabolomics Analysis of Giant Embryo Development in Rice
Source: Front Plant Sci. 2021 Aug 9;12:697889. doi: 10.3389/fpls.2021.697889 (PMC8381154; doi:10.3389/fpls.2021.697889)
Supplement: Supplementary file 2 [file Data_Sheet_2.docx]

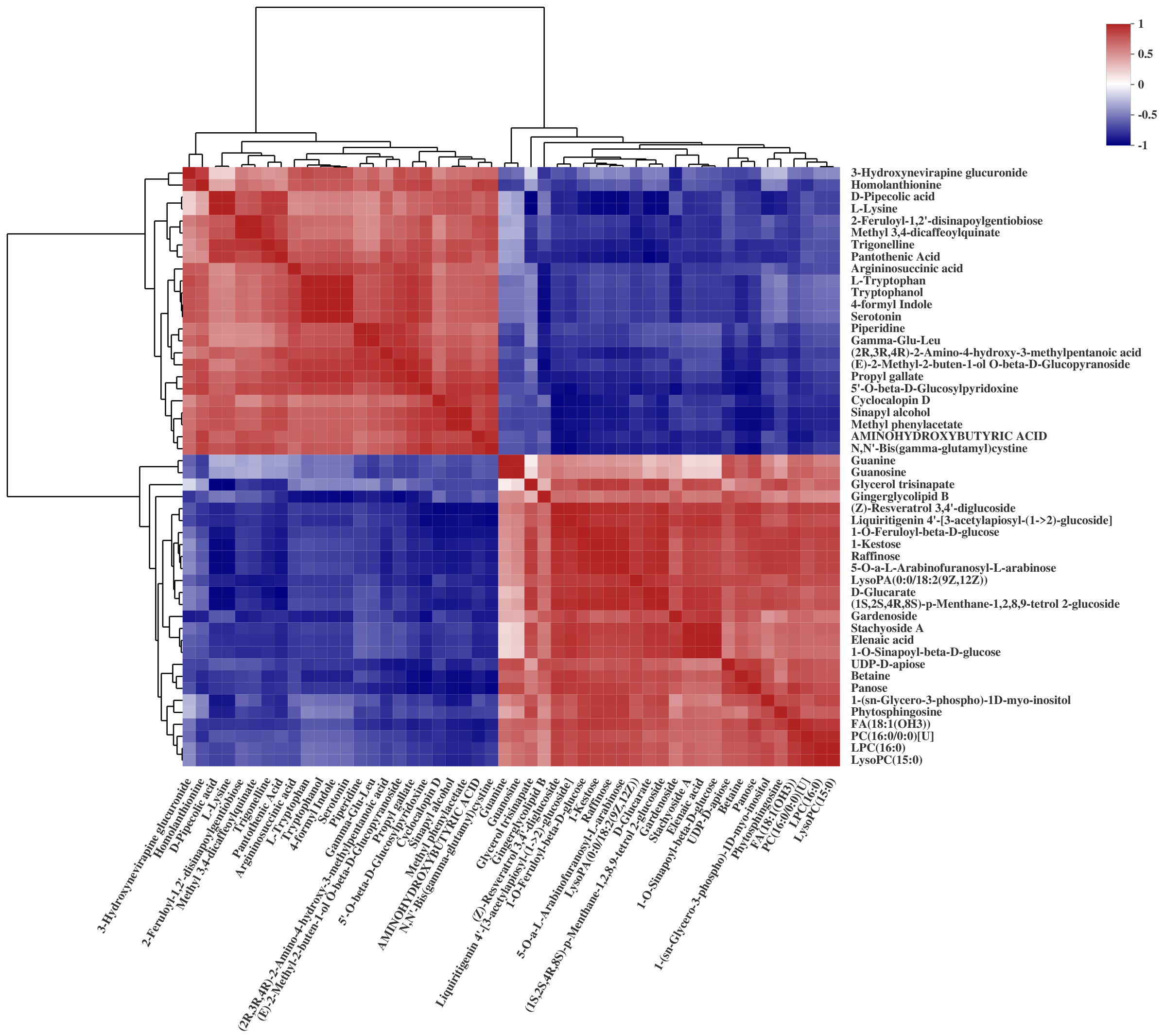


**Figure S2.** Top fifty metabolite correlation analysis. The name of the right and below are the metabolites, the left and above are the metabolite cluster tree map, different colors represent the size of the correlation coefficient, the correlation coefficient is positive, positive and negative values indicate the positive and negative correlation, the closer the absolute value is to 1, the higher the positive or negative correlation of the metabolites.
