# Supplementary material for: Identification of a New Giant Emrbryo Allele, and Integrated Transcriptomics and Metabolomics Analysis of Giant Embryo Development in Rice
Source: Front Plant Sci. 2021 Aug 9;12:697889. doi: 10.3389/fpls.2021.697889 (PMC8381154; doi:10.3389/fpls.2021.697889)
Supplement: Supplementary file 3 [file Data_Sheet_3.docx]

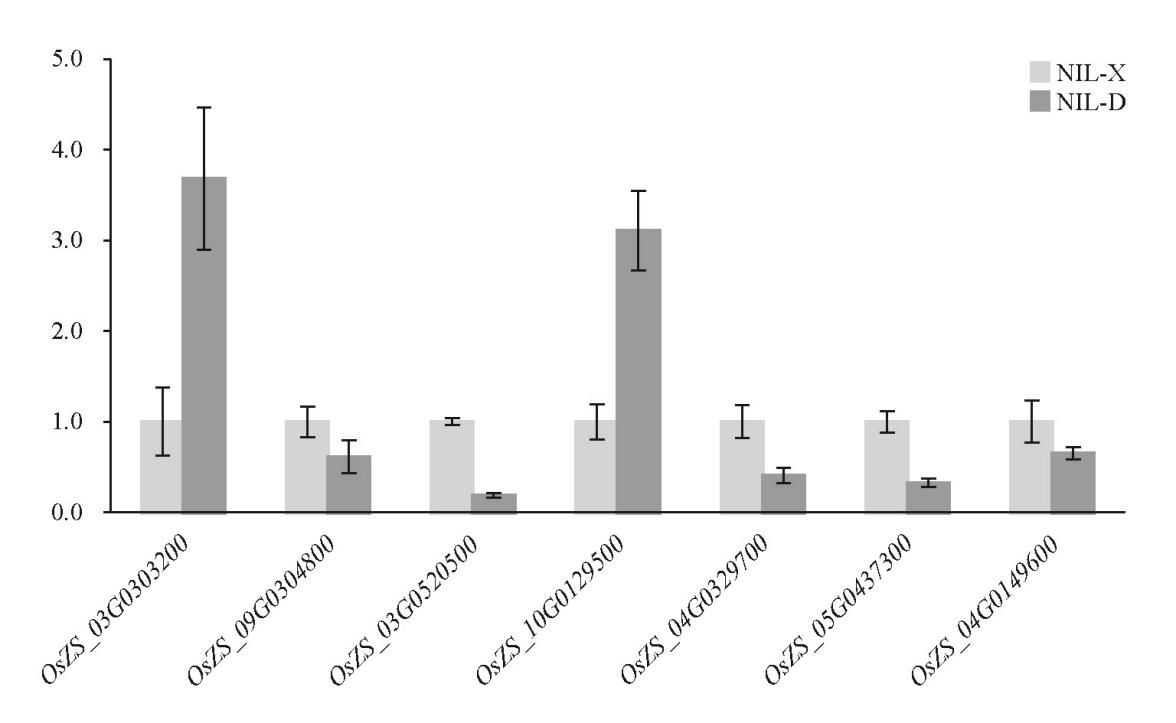


**Figure S3.** Validation of RNA-Seq Data by RT-qPCR. Seven candidate genes associated with amino acid metabolism, energy metabolism and lipid metabolism pathways were selected and their expressions in the 187R wild-type (NIL-X) and Dapeimi mutant-type (NIL-D) were checked with RT-qPCR. The data are shown as means ± s.e.m. (n=3).
