# Supplementary material for: Identification of a New Giant Emrbryo Allele, and Integrated Transcriptomics and Metabolomics Analysis of Giant Embryo Development in Rice
Source: Front Plant Sci. 2021 Aug 9;12:697889. doi: 10.3389/fpls.2021.697889 (PMC8381154; doi:10.3389/fpls.2021.697889)
Supplement: Supplementary file 4 [file Data_Sheet_4.docx]

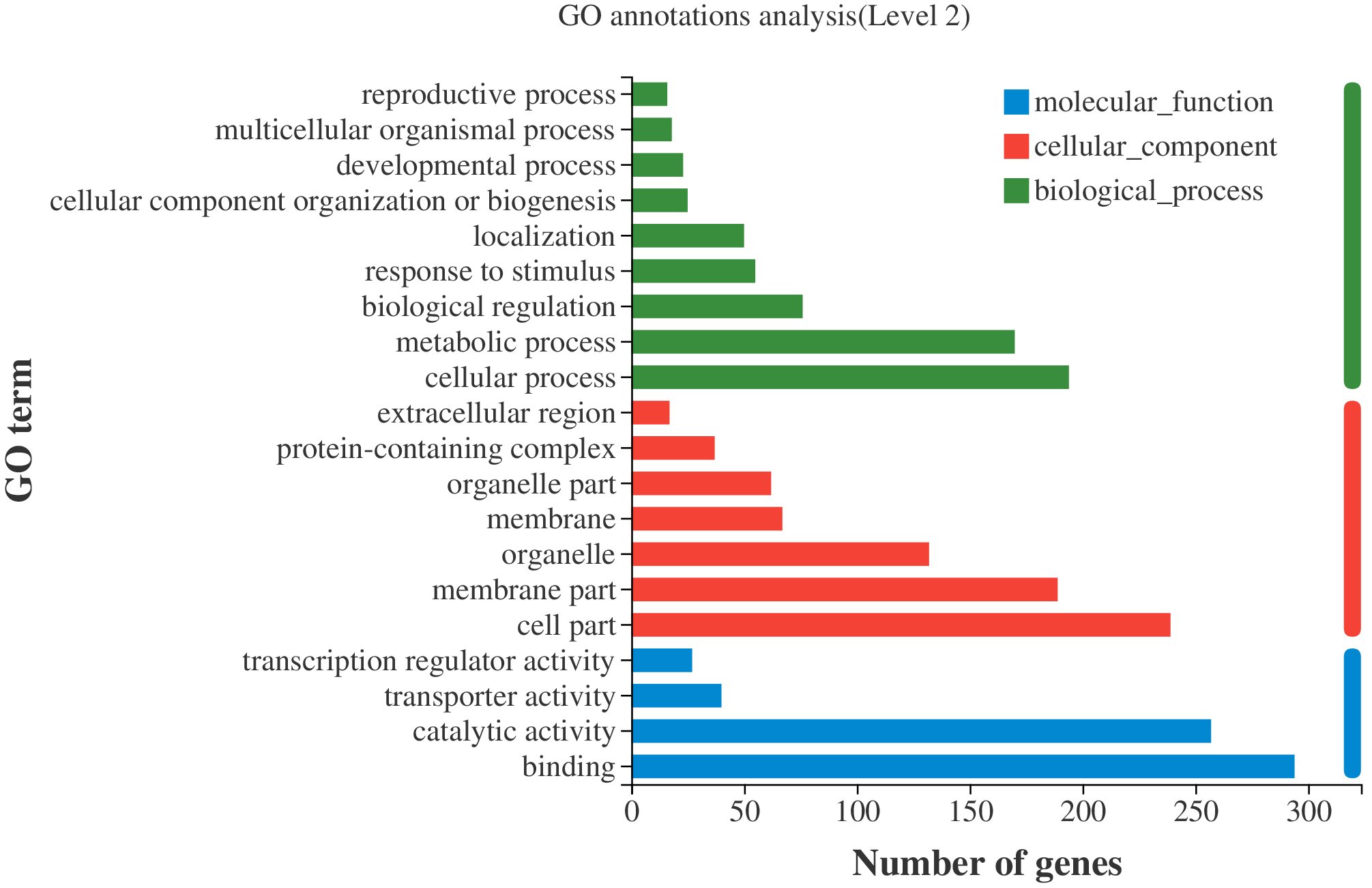


**Figure S4.** GO analysis. A vertical coordinate represents terms of secondary classification, transverse coordinates represent the number of genes/transcripts of secondary classification, three colors represent molecular function, cellular components and biological processes.
