# Supplementary material for: Identification of a New Giant Emrbryo Allele, and Integrated Transcriptomics and Metabolomics Analysis of Giant Embryo Development in Rice
Source: Front Plant Sci. 2021 Aug 9;12:697889. doi: 10.3389/fpls.2021.697889 (PMC8381154; doi:10.3389/fpls.2021.697889)
Supplement: Supplementary file 5 [file Table_1.DOCX]

**Table S1 Primers used for the study**

| Name | Locus | Forward primer (5’-3’) | Reverse primer (5’-3’) | Purpose |
| --- | --- | --- | --- | --- |
| RM1365 | NA | CAGGCGGTGATTTTCTTCTC | GACAACTCAGTTCCAATCCC | Mapping |
| RM1132 | NA | ATCACCTGAGAAACATCCGG | CTCCTCCCACGTCAAGGTC | Mapping |
| RM21931 | NA | CCTGCATTTCCTTTCAACTCATGG | GTGTTCTTGTGATCGTGCTCTCG | Mapping |
| RM21938 | NA | CCAAATTGCTTCCTCGGATATAGG | CGGATTTAGGGAGTTCGTGTTCG | Mapping |
| RM7441 | NA | CAGGCAATTCTGTTGTGGTG | GGGAGAAGCATCCAAGGAAG | Mapping |
| RM18 | NA | TTCCCTCTCATGAGCTCCAT | GAGTGCCTGGCGCTGTAC | Mapping |
| DST900-1 | OsZS_07T0416900 | TTCGCGAACGCTTCCCAT | GCCGAAATGCGGTTTGTGAT | Direct sequencing |
| DST900-2 | OsZS_07T0416900 | ACGTCGCCTCCCTCATGTCC | CTGGATGTAGGGGAGGCTCG | Direct sequencing |
| GE-OEX | OsZS_07T0416900 | AACACGGGGGACTCTTGACCATGGCGCTCTCCTCCATGGC | CGGGGAAATTCGAGCTGGTCACCTCAGGCCCTAGCCACGGCC | Over-expression |
| NA | OsZS_03G0303200 | ACGACACGGCGTACCGATTT | TGGGTTCCTGGGCACAAGC | qRT-PCR |
| NA | OsZS_09G0304800 | AAGGATCAGGAGCCGACGGA | CAAAGAAGACGGCGACGAGG | qRT-PCR |
| NA | OsZS_03G0520500 | GCACTCATAGGCTCACAACTCAG | CTCCTCCCAGACACTTCTATCAA | qRT-PCR |
| NA | OsZS_10G0129500 | AGGAAGCAAAGCTCCTGACACG | GCTTAAAGGTGGAGTTTGGACGC | qRT-PCR |
| NA | OsZS_04G0329700 | GCGGAAGAGGACAGTGCACA | ATGGTGTTCGCAGCTGTGTAGC | qRT-PCR |
| NA | OsZS_05G0437300 | TACATAGAGCAGAGCGGTTACTATC | ATGACGCCATCAAAGACACCAC | qRT-PCR |
| NA | OsZS_04G0149600 | TCATCTCATCTGGAGGAGTGGC | TCATTGGTCTGGCGATCTTTG | qRT-PCR |
