# Supplementary material for: Identification of a New Giant Emrbryo Allele, and Integrated Transcriptomics and Metabolomics Analysis of Giant Embryo Development in Rice
Source: Front Plant Sci. 2021 Aug 9;12:697889. doi: 10.3389/fpls.2021.697889 (PMC8381154; doi:10.3389/fpls.2021.697889)
Supplement: Supplementary file 6 [file Table_2.docx]

**Table S2 Annotation analysis of the transcript function database**

|  | Expre_Gene number (percent) | Expre_Transcript number (percent) | All_Gene number (percent) | All_Transcript number (percent) |
| --- | --- | --- | --- | --- |
| GO | 25703(78.32%) | 40151(81.1%) | 43627(71.64%) | 63939(75.26%) |
| KEGG | 9765(29.76%) | 17845(36.04%) | 11937(19.6%) | 22521(26.51%) |
| COG | 26693(81.34%) | 42979(86.81%) | 39873(65.48%) | 62332(73.37%) |
| NR | 31997(97.5%) | 48720(98.4%) | 58024(95.28%) | 81880(96.38%) |
| Swiss-Prot | 21055(64.16%) | 35155(71.01%) | 28147(46.22%) | 47432(55.83%) |
| Pfam | 24063(73.32%) | 38122(77%) | 35327(58.01%) | 54728(64.42%) |
| Total_anno | 32023(97.58%) | 48744(98.45%) | 58083(95.38%) | 81945(96.45%) |
| Total | 32817(100%) | 49510(100%) | 60897(100%) | 84957(100%) |
