# Supplementary material for: Identification of a New Giant Emrbryo Allele, and Integrated Transcriptomics and Metabolomics Analysis of Giant Embryo Development in Rice
Source: Front Plant Sci. 2021 Aug 9;12:697889. doi: 10.3389/fpls.2021.697889 (PMC8381154; doi:10.3389/fpls.2021.697889)
Supplement: Supplementary file 12 [file Table_8.DOCX]

**
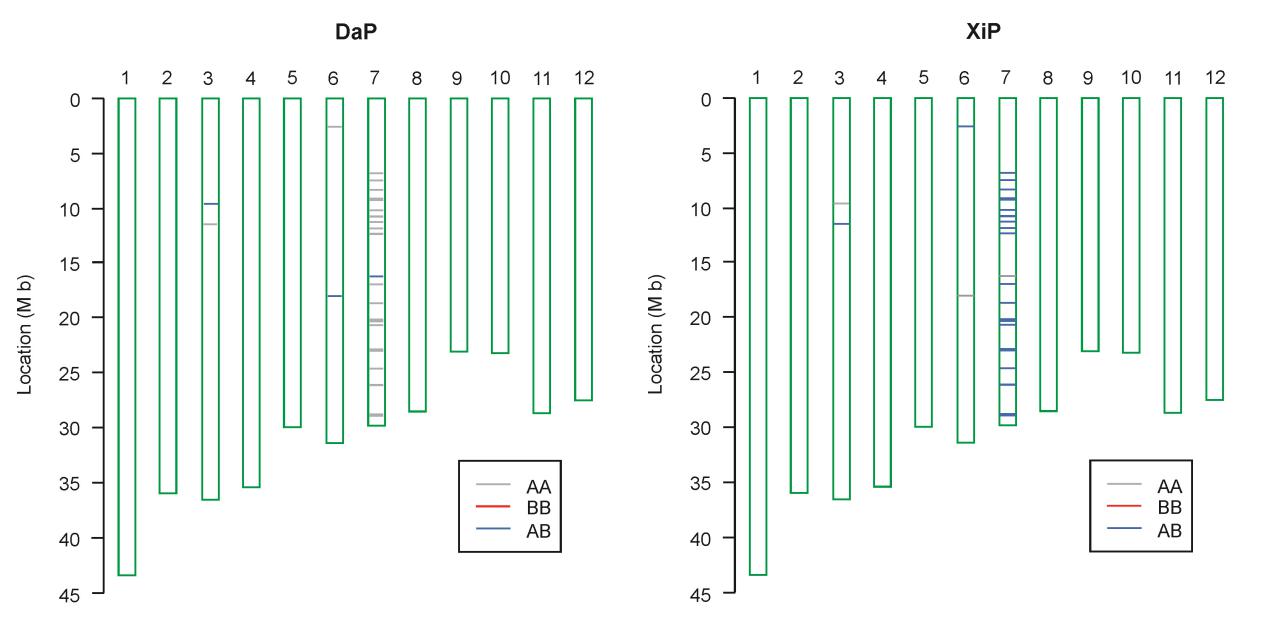
**

Fig. S1 SNP chip localization using BSA mixed pool analysis. The giant embryo and normoal embryo genotype are indicated by letters AA and BB separately, AB represents heterozygous genotype.

**
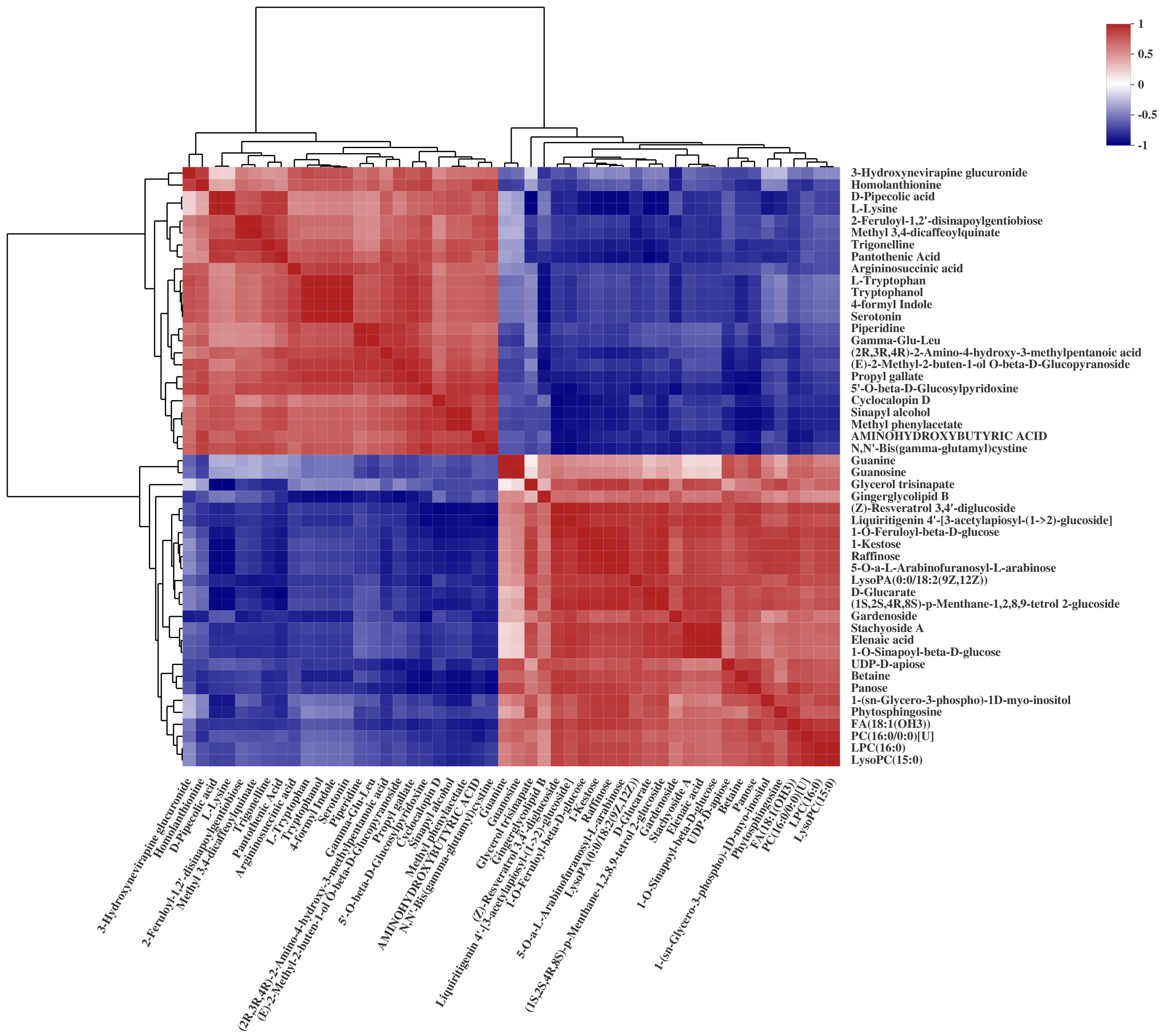
**

Fig. S2 Top 50 metabolite correlation analysis. The name of the right and below are the metabolites, the left and above are the metabolite cluster tree map, different colors represent the size of the correlation coefficient, the correlation coefficient is positive, positive and negative values indicate the positive and negative correlation, the closer the absolute value is to 1, the higher the positive or negative correlation of the metabolites.

**
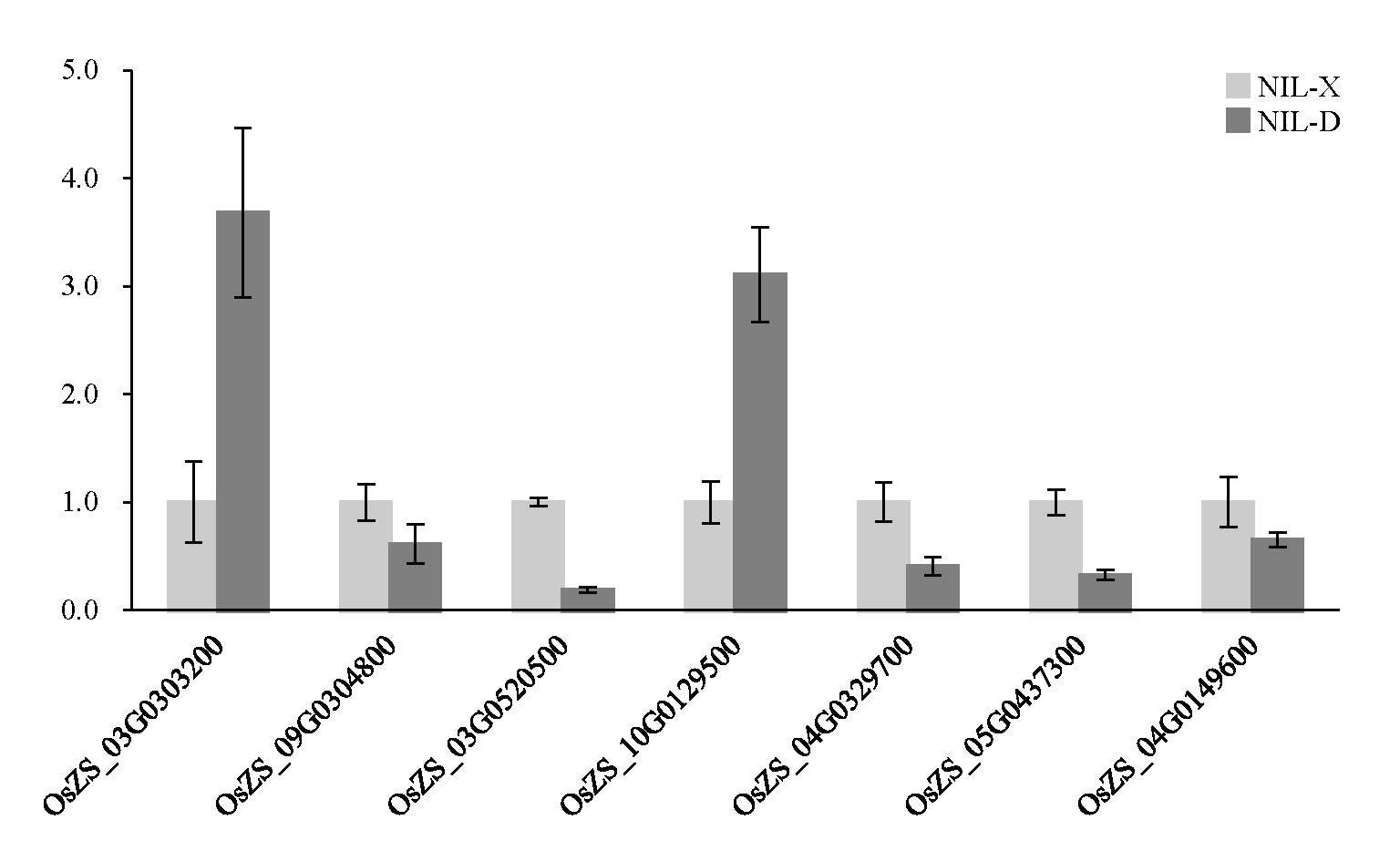
**

Fig. S3 Validation of RNA-Seq Data by qRT-PCR. Seven candidate genes associated with amino acid metabolism, energy metabolism and lipid metabolism pathways were selected and their expressions in the 187R wild-type (NIL-X) and Dapeimi mutant-type (NIL-D) were checked with qRT-PCR. The data are shown as means ± s.e.m. (n=3).


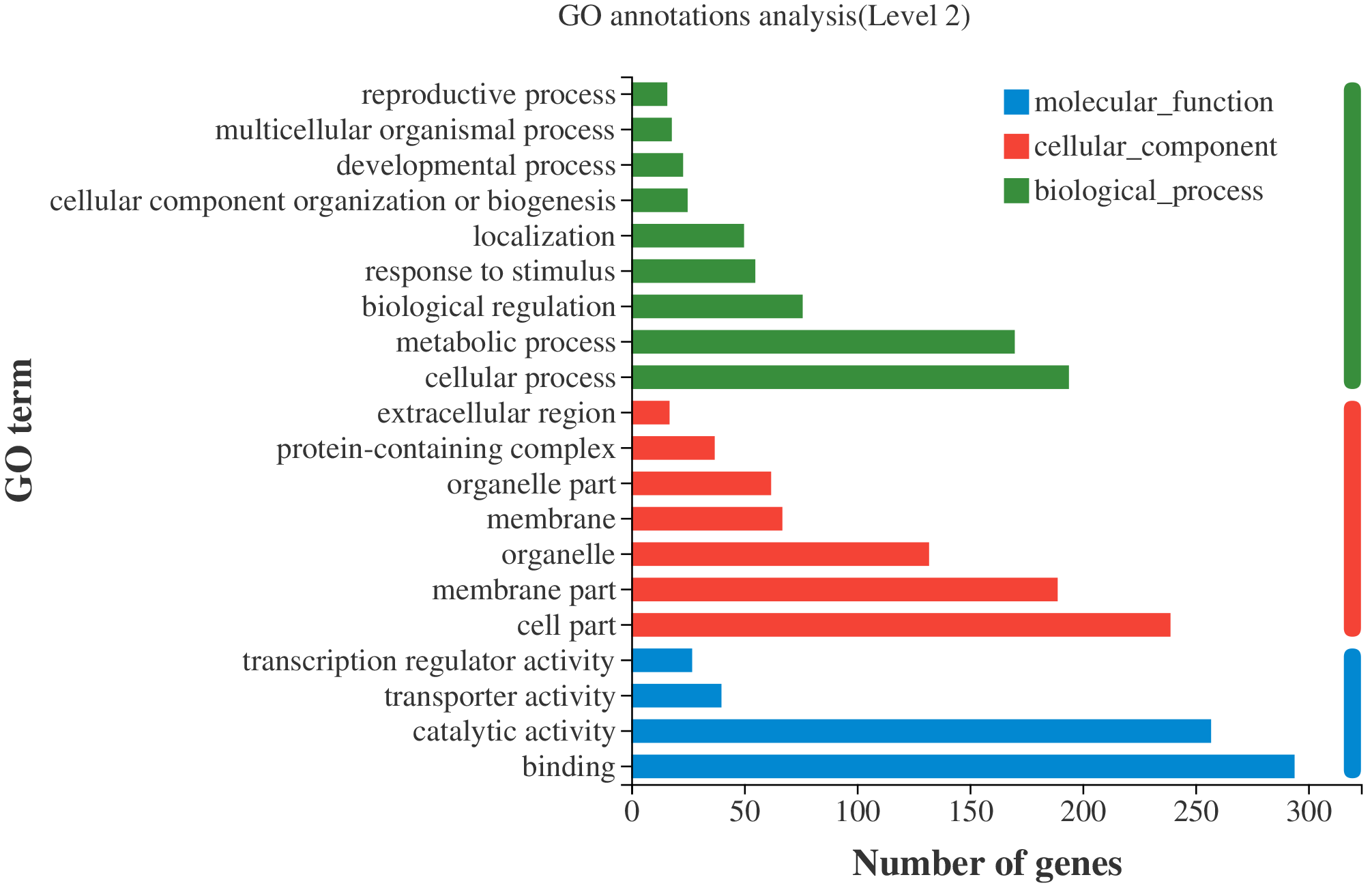


Fig. S4 GO analysis. A vertical coordinate represents terms of secondary classification, transverse coordinates represent the number of genes/transcripts of secondary classification, three colors represent molecular function, cellular components and biological processes.
